# The relationship between the systemic inflammatory response and survival in patients with transitional cell carcinoma of the urinary bladder

**DOI:** 10.1038/sj.bjc.6602406

**Published:** 2005-02-22

**Authors:** M Hilmy, J M S Bartlett, M A Underwood, D C McMillan

**Affiliations:** 1 University Department of Surgery, Royal Infirmary, Glasgow G31 2ER, UK

**Keywords:** bladder cancer, stage, grade, C-reactive protein, survival

## Abstract

The relationship between tumour stage, grade, elevated C-reactive protein concentration (<10/>10 mg l^−1^), adjuvant therapy and survival was examined in patients with biopsy proven bladder cancer (*n*=105). On multivariate analysis stage (HR 3.37, 95% CI 1.37–8.29, *P*=0.008), grade (HR 2.01, 95% CI 1.14–3.57, *P*=0.017) and preoperative C-reactive protein (HR 3.31, 95% CI 1.09–10.09, *P*=0.035) were independently associated with cancer-specific survival.

Bladder cancer is the fourth most common malignancy in United Kingdom in men and the eighth in women with a total of 12 500 new cases each year causing 5000 deaths annually (CRC CancerStats, 2002). More than 90% of bladder cancers diagnosed in the Western world, are transitional cell carcinomas and constitute a significant proportion of the general urological consultants' workload due to its high prevalence and recurrent nature.

It is increasingly recognised that, in addition to tumour stage, disease progression is dependent on a complex interaction of the tumour and host inflammatory response (Balkwill and Mantovani, 2001; Coussens and Werb, 2002). Indeed, the systemic inflammatory response, as evidenced by elevated circulating concentrations of C-reactive protein, has been shown to be a stage independent prognostic factor in a variety of operable tumours (Mahmoud and Rivera, 2002; Ikeda *et al*, 2003; McMillan *et al*, 2003). To our knowledge there has been only one previous study that has examined the prognostic value of C-reactive protein in patients with invasive bladder cancer (O'Quigley *et al*, 1981).

The aim of the present study was to examine the relationship between stage, grade, C-reactive protein and cancer-specific survival in patients with superficial or invasive bladder cancer.

## PATIENTS AND METHODS

Patients with biopsy proven transitional cell carcinoma presenting to a single urological unit at Glasgow Royal Infirmary between 1992 and 1999 were reviewed. Those patients with a measurement of C-reactive protein prior to or 3–6 months following transurethral resection of bladder tumour were included in the study as previously described (McMillan *et al*, 2003). Tumour stage was assessed using the UICC guidelines, and tumour grade performed according to the WHO grading system. Date and cause of death were obtained from the Cancer Registry, Scotland using ICD 9 code (bladder cancer 1889).

Routine laboratory measurement of patient's serum for C-reactive protein concentration was performed. The limit of detection of the assay was a C-reactive protein concentration lower than 5 mg l^−1^. The coefficient of variation, over the range of measurement, was less than 5% as established by routine quality control procedures. C-reactive protein measurement of greater than 10 mg l^−1^ was considered to indicate the presence of systemic inflammatory response. (O'Gorman *et al*, 2000).

The Research Ethics Committee of North Glasgow NHS Trust approved the study.

## STATISTICAL ANALYSIS

Survival analysis was performed using the Cox proportional hazard model with patient age, sex, tumour stage and grade and C-reactive protein concentration as prognostic variables. Deaths up to September 2004 were included in the analysis.

Multivariate survival analysis was performed using stepwise backward procedure to derive a final model of the variables that had a significant independent relationship with survival. To remove a variable from the model, the corresponding *P*-value had to be a greater than 0.10. Analysis was performed using SPSS software (SPSS, Chicago, IL, USA).

## RESULTS

The characteristics of patients with bladder cancer (*n*=105) are shown in Table 1. The majority were male, over the age of 65 years, had superficial disease (Ta, T1) and had elevated C-reactive protein concentrations either pre- or postoperatively. In total, 24 patients had adjuvant therapy (eight radiotherapy, eight BCG, eight cystectomy).

In all, 32 patients had C-reactive protein concentrations determined both prior to and following the resection. Of the 20 patients with an elevated preoperative value, 12 (60%) had a raised concentration after the operation. Of the 12 patients with a normal preoperative value, nine (75%) had a postoperative value in the normal range.

During the follow-up period 80 patients died, 41 of them from bladder cancer and the mean cancer-specific survival was 82.9 months (95% confidence interval 70.8–94.9). On univariate analysis, stage (*P*<0.001), grade (*P*<0.01) and elevated C-reactive protein preoperatively (*P*<0.05) and postoperatively (*P*<0.01) were significantly associated with overall survival. Also, stage (*P*<0.001), grade (*P*<0.001) and elevated C-reactive protein preoperatively (*P*<0.05) and postoperatively (*P*<0.01) were significantly associated with cancer-specific survival (Table 2).

On multivariate analysis of patients who had a preoperative C-reactive protein determination, stage (HR 4.00, 95% CI 2.02–7.94, *P*<0.001) and preoperative C-reactive protein (HR 2.73, 95% CI 1.23–6.07, *P*=0.014) were independently associated with overall survival. Also, stage (HR 3.37, 95% CI 1.37–8.29, *P*=0.008), grade (HR 2.01, 95% CI 1.14–3.57, *P*=0.017) and preoperative C-reactive protein (HR 3.31, 95% CI 1.09–10.09, *P*=0.035) were independently associated with cancer-specific survival. Those patients with an elevated preoperative C-reactive protein concentration (>10 mg l^−1^) had a mean cancer-specific survival of 65.5 months (95% CI 46.8–84.2) compared with 103.7 months (95% CI 81.8–125.6) in those patients with a C-reactive protein concentration in the normal range (⩽10 mg l^−1^).

## DISCUSSION

In the present study, both tumour grade and the presence of a systemic inflammatory response, as evidenced by elevated circulating concentrations of C-reactive protein, were independent predictors of cancer-specific survival in patients with bladder cancer. These results confirm the importance of tumour grade as a stage independent prognostic factor (Ali-El-Dein *et al*, 2003; Nishiyama *et al*, 2004) and are consistent with the work of O'Quigley *et al* (1981), who reported that an elevated C-reactive protein concentration, measured by radial immunodiffusion and using the limit of sensitivity of 12 mg l^−1^ as a cutoff, was associated with poor survival in patients with invasive bladder cancer.

Furthermore, because C-reactive protein concentration is independent of tumour stage and grade, the presence or absence of a systemic inflammatory response, might, form the basis of a new prognostic score that reflects not only the tumour but also the host response. Indeed, this approach has recently been used in both lung and colorectal cancer (Forrest *et al*, 2003; Canna *et al*, 2004).

Although in the present study, it may appear from the present results that an elevated postoperative C-reactive protein concentration is a better predictor of cancer-specific survival than an elevated concentration prior to resection, in a clinical context the measurement of preoperative CRP concentration is likely to be of more value because it would allow planning of adjuvant therapy.

The mechanisms by which a systemic inflammatory response might impact on cancer-specific survival are not clear. However, it is known that as part of the systemic inflammatory response to the tumour there is a release of proinflammatory cytokines and growth factors, which not only stimulate tumour growth (Abramovitch *et al*, 1999; Coussens and Werb, 2002) but also produce profound catabolic effects on host metabolism (Kotler, 2000). For example, interleukin-6, produced by the tumour or infiltrating inflammatory cells, is recognised as a growth promoter in bladder cancer (Okamoto *et al*, 1997; Andrews *et al*, 2002). Interleukin-6 also stimulates liver production of acute-phase proteins, such as C-reactive protein, which increases the demand for certain amino acids, which, if limited in the diet, must be obtained from the breakdown of skeletal muscle (McMillan *et al*, 1998; Preston *et al*, 1998). In this way, the presence and magnitude of a chronic systemic inflammatory response may produce a progressive nutritional and functional decline, ultimately resulting in reduced survival.

In summary, it would appear that the systemic inflammatory response is a stage and grade independent prognostic factor in patients with bladder cancer. The role of the systemic inflammatory response in determining disease-specific survival in patients with bladder cancer is worthy of further study to establish its value as a prognostic factor.

## Figures and Tables

**Table 1:** Clinical characteristics and survival in patients with bladder cancer

	**Patients**
	**105 (100%)**
*Age*	
⩽65 years	37 (35)
>65 years	68 (65)
*Sex*	
Male	75 (71)
Female	30 (29)
*Stage*	
Superficial (Ta,T1)	76 (72)
Invasive	29 (28)
*Grade*	
G1	36 (34)
G2	31 (30)
G3	38 (36)
*Preoperative C-reactive protein*	
⩽10 mg l^−1^	16 (15)
>10 mg l^−1^	43 (41)
*Postoperative C-reactive protein*	
⩽10 mg l^−1^	36 (34)
>10 mg l^−1^	42 (40)
*Adjuvant therapy*	
No	81 (77)
Yes	24 (23)
Alive	25 (24)
*Dead*	
Bladder cancer specific	41 (39)
Intercurrent	39 (37)

**Table 2:** The relationship between variables and overall and cancer-specific survival in patients with bladder cancer: univariate analysis

	**Overall**		**Cancer specific**	
	**Hazard ratio**	***P*-value^a^**	**Hazard ratio**	***P*-value^a^**
Age (⩽65/>65 years)		0.063		0.142
Sex (male/female)		0.338		0.803
*Stage*				
(Superficial/invasive)	2.82 (1.74–4.57)	<0.001	4.96 (2.61–9.41)	<0.001
Grade (G1/G2/G3)	1.48 (1.14–1.92)	0.004	2.17 (1.45–3.23)	<0.001
Preoperative C-reactive protein (⩽10/>10 mg l^−1^)	2.50 (1.15–5.43)	0.021	3.03 (1.04–8.87)	0.043
Postoperative C-reactive protein (⩽10/>10 mg l^−1^)	2.31 (1.34–3.97)	0.003	3.28 (1.41–7.63)	0.006
Adjuvant therapy (No/Yes)		0.537		0.732

Values in parentheses are 95% confidence intervals.

a Cox proportional hazard model.
